# Intravenous ferric carboxymaltose in patients with heart failure and iron deficiency: a systematic review and meta-analysis of randomized controlled trials with trial sequential analysis

**DOI:** 10.1093/eschf/xvaf018

**Published:** 2026-01-08

**Authors:** Mushood Ahmed, Eeshal Zulfiqar, Tallal Mushtaq Hashmi, Rohma Zia, Hadiah Ashraf, Muhammad Abdullah Naveed, Raheel Ahmed, Jamal S Rana, Faizan Ahmed, Stephen J Greene, Marat Fudim, Robert J Mentz, Gregg C Fonarow

**Affiliations:** Department of Cardiology, Rawalpindi Medical University, Rawalpindi, Pakistan; Department of Cardiology, Dow University of Health Sciences, Karachi, Pakistan; Department of Cardiology, Rawalpindi Medical University, Rawalpindi, Pakistan; Department of Cardiology, Rawalpindi Medical University, Rawalpindi, Pakistan; Department of Cardiology, Rawalpindi Medical University, Rawalpindi, Pakistan; Department of Cardiology, Dow University of Health Sciences, Karachi, Pakistan; Department of Cardiology, Royal Brompton Hospital, London UK; National Heart and Lung Institute, Imperial College London, London, UK; Division of Cardiology, Kaiser Permanente Northern California, Oakland, CA, USA; Division of Cardiology, Duke University Medical Center, Durham, NC, USA; Division of Cardiology, Duke University Medical Center, Durham, NC, USA; Division of Cardiology, Duke Clinical Research Institute, Durham, NC, USA; Division of Cardiology, Duke University Medical Center, Durham, NC, USA; Division of Cardiology, Duke Clinical Research Institute, Durham, NC, USA; Division of Cardiology, Duke University Medical Center, Durham, NC, USA; Division of Cardiology, Duke Clinical Research Institute, Durham, NC, USA; Ahmanson-UCLA Cardiomyopathy Center, Division of Cardiology, University of California Los Angeles, Los Angeles, CA, USA

**Keywords:** Heart failure, Iron deficiency, Ferric carboxymaltose, Meta-analysis

## Abstract

**Introduction:**

Iron deficiency (ID) is common among patients with heart failure (HF), and it is associated with poor functional outcomes, increased hospitalizations, and higher mortality. This meta-analysis evaluates the efficacy of intravenous ferric carboxymaltose (FCM) in HF patients with ID.

**Methods:**

We conducted a literature search of major bibliographic databases up to 15 April 2025, to identify randomized controlled trials (RCTs) comparing FCM with placebo or standard care in HF patients with ID. The primary outcome was a composite of recurrent hospitalizations for heart failure (HHF) or cardiovascular (CV) death assessed at 1-year and complete follow-up. Risk ratios (RR) and mean differences (MD) with 95% confidence intervals (CI) were estimated using a random-effects model.

**Results:**

Eleven RCTs enrolling 6493 patients (3329 FCM; 3164 control) were included. The mean age of patients was 66.7 ± 10.6 years, 34.4% were women, mean left ventricular ejection fraction was 33.7 ± 8.8%, mean haemoglobin was 12.4 ± 1.8 g/dL, and mean transferrin saturation was 18.9 ± 10.1%. FCM significantly reduced the composite of recurrent HHF or CV death at 1-year (RR 0.73, 95% CI 0.62–0.85) and over maximum follow-up (RR 0.80, 95% CI 0.68–0.94) compared to control. Recurrent HHF was significantly reduced with FCM administration (1-year RR 0.69, 95% CI 0.57–0.84; complete follow-up RR 0.75, 95% CI 0.60–0.94). FCM demonstrated a trend towards reduced all-cause (RR: 0.86, 95% CI: 0.74–1.00) and CV mortality at 1-year (RR: 0.86, 95% CI: 0.72–1.02), but this effect was attenuated over longer follow-up. FCM significantly improved 6-minute walk test performance (MD 29.19 m, 95% CI 11.95–46.43). The trial sequential analysis confirmed robust evidence for the primary outcome.

**Conclusion:**

Intravenous FCM in HF patients is associated with reduced risk of adverse cardiovascular events and improved functional capacity. Further trials are needed to clarify its long-term survival impact.

## Graphical Abstract

**Graphical Abstract:**
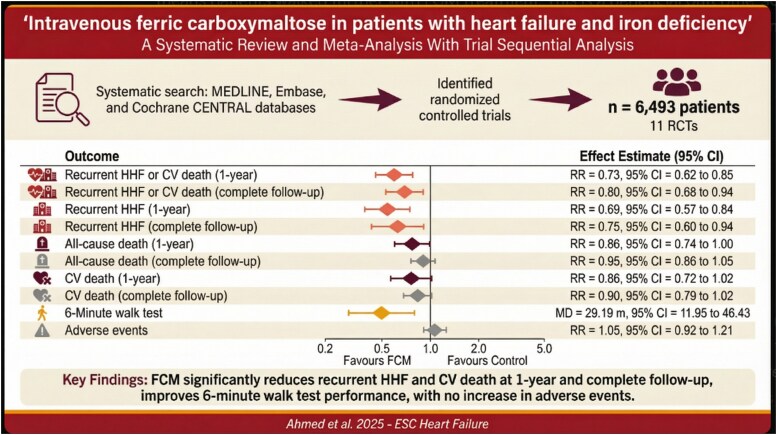

## Introduction

Iron deficiency (ID) affects approximately 50% of patients with heart failure (HF), and is recognized as a significant comorbidity in this population.^1,2^ Although often associated with anaemia, the two conditions do not necessarily coexist, and both have been shown to independently worsen outcomes in patients with HF.^3^ Iron is crucial for oxygen transport and cellular energy production, and thus, its deficiency leads to reduced oxygen delivery and utilization, impairing cardiac and skeletal muscle function.^4^ Iron deficiency is associated with poor functional outcomes, including reduced exercise capacity and worse quality of life, leading to increased hospitalization rates and higher mortality risk.^5–8^ The underlying causes of ID in HF remain incompletely understood, involving mechanisms such as impaired absorption and inflammation.^4^ Independent predictors of ID include female sex, advanced age, and more severe NYHA class, which are also associated with increased healthcare utilization and costs.^6,9^

Current guidelines from the European Society of Cardiology and the American Heart Association/American College of Cardiology/Heart Failure Society of America recommend regular screening for ID in this population. For patients with HF with reduced ejection fraction (HFrEF) and confirmed ID, both guidelines support intravenous iron therapy—albeit with different levels of recommendation for QOL improvement. These recommendations are based on evidence demonstrating improvements in symptoms, exercise tolerance, and quality of life, with the ESC guidelines further suggesting a potential reduction in heart failure hospitalizations (HHF).^10,11^

Several studies have consistently demonstrated that intravenous iron therapy with ferric carboxymaltose (FCM) leads to significant improvements in exercise endurance, symptoms, and quality of life among patients with HF and ID.^12,13^ However, its impact on mortality and long-term clinical outcomes remains debated. While prior meta-analyses have yielded conflicting results regarding the clinical efficacy of FCM,^14,15^ our study seeks to advance the current evidence base by systematically synthesizing all trials published to date. Moreover, in addition to conventional relative effect measures, we calculated absolute risk reductions, performed trial sequential analysis to evaluate the robustness of findings, applied the GRADE framework to assess the certainty of evidence, and examined a broader range of clinically relevant outcomes.

## Methods

This systematic review and meta-analysis was conducted in accordance with the Preferred Reporting Items for Systematic Reviews and Meta-Analyses (PRISMA) guidelines.^16^ The study protocol was registered with PROSPERO (CRD420251034475).

### Data sources and search strategy

Two investigators independently performed systematic searches of PubMed/MEDLINE, Embase, and the Cochrane Library for randomized controlled trials examining the efficacy and safety of FCM in patients with HF. The search encompassed all available records from the inception of each database until 15 April 2025. Additionally, manual searches of reference lists from relevant articles and previous meta-analyses were conducted to identify any additional eligible studies. The search strategy incorporated a combination of keywords and Medical Subject Headings terms, including variations of ‘ferric carboxymaltose,’ ‘FCM,’ ‘iron supplementation,’ ‘heart failure,’ and ‘HF.’ The complete search strategies for each database are provided in Supplementary Table S1.

### Study selection, eligibility criteria and outcomes

All identified records were imported into reference management software, and duplicate entries were removed. Two reviewers independently screened the titles and abstracts of all retrieved articles, excluding those that clearly did not meet the inclusion criteria. The full texts of potentially eligible studies were then assessed in detail. Any disagreements regarding study eligibility were resolved through discussion or by consulting a third reviewer. Studies were included if they met the following criteria: they were randomized controlled trials with a minimum follow-up period of two months; they enrolled adult patients aged 18 years or older with a diagnosis of HF; they compared FCM with either placebo or standard care; and they reported data on at least one predefined clinical outcome.

The primary outcome of interest was a composite of recurrent HHF and cardiovascular (CV) death. The secondary outcomes included recurrent HHF, all-cause death, CV death, the 6-minute walk test (6-MWT), and adverse events. The published individual patient data (IPD) reports of included trials were also reviewed to ensure data extraction for missing data in study-level publications. Studies were excluded if they were non-randomized, lacked a control group, or did not provide sufficient outcome data. There were no restrictions based on language or publication date.

### Data extraction

Two reviewers independently extracted relevant data from each included study using a standardized form. The extracted information included study characteristics such as author names, publication year, study design, sample size, and follow-up duration. Patient demographics and clinical characteristics, including age, sex, and baseline iron status, were also recorded. For outcomes, data on event rates and effect estimates were collected. A third reviewer verified the accuracy and completeness of the extracted data.

### Bias and certainty of evidence

The methodological quality of the included randomized controlled trials was assessed using the Cochrane Risk of Bias Tool for Randomized Trials (RoB 2.0).^17^ This tool evaluates potential biases across several domains, including the randomization process, deviations from intended interventions, missing outcome data, outcome measurement, and selective reporting. Based on these assessments, studies were categorized as having a low risk of bias, some concerns, or a high risk of bias. Two authors independently evaluated the certainty of evidence for each outcome using the GRADE approach, which considers factors such as risk of bias, inconsistency, indirectness, imprecision, and publication bias. The certainty of evidence was categorized as high, moderate, low, or very low. Any disagreements were resolved through consultation with a third reviewer.

### Statistical analysis

All statistical analyses were performed using R software (version 4.3.3) with the ‘meta’ and ‘metasens’ packages. Risk ratios with 95% confidence intervals were pooled for dichotomous outcomes using a random-effects model.^18^ For continuous outcomes, weighted mean differences (WMD) were pooled. Heterogeneity was estimated using the Paule-Mandel estimator, and its magnitude was assessed using the I^2^ statistic, with values above 60% indicating substantial heterogeneity, respectively.^19^ Absolute risk differences were estimated by applying pooled RRs to the baseline risk from the control groups. Summary of findings tables were generated using GRADEpro (https://gdt.gradepro.org/app/).

Trial Sequential Analysis (TSA) is a methodological tool utilized in meta-analyses to evaluate the conclusiveness and reliability of accumulated evidence from RCTs. It incorporates the principles of sequential monitoring from interim analyses to mitigate the risk of Type I and Type II errors, which are particularly prevalent in meta-analyses involving a limited number of trials or small sample sizes. TSA calculates the required information size, the meta-analytical equivalent of the sample size in a single trial, and applies monitoring boundaries to assess whether the cumulative data provide sufficient evidence for a definitive conclusion. If the cumulative Z-curve surpasses these boundaries, it suggests that the evidence is statistically sound. This approach enhances the validity of meta-analytic findings by reducing the likelihood of premature conclusions.

The TSA computations, including information size estimation and boundary delineation, were performed using the Copenhagen Trial Unit’s Centre for Clinical Intervention Research software package (version 0.9.5.10 Beta).^20^ TSA was performed for composite HHF or CVD, recurrent HHF, CV death, and all-cause death, assuming a 25% relative risk reduction, an alpha level of 5%, and 90% power. A relative risk reduction of 25% was chosen to represent a reasonable clinical intervention effect based on recent clinical trials [FAIR-HF2 and HEART-FID trials]. The proportion of events in the control group was estimated from the control groups included in the meta-analysis. All statistical tests were two-sided, and a *P*-value of less than 0.05 was considered statistically significant.

## Results

A total of 1150 studies were identified in the initial literature search. After screening of articles against the predefined eligibility criteria, 11 RCTs^12,13,21–29^ were included in the meta-analysis (Supplementary Fig. S1). The PRIMSA checklist is reported as Supplementary Table S2.

The included studies enrolled a total of 6493 patients, with 3329 randomized to FCM (FCM) and 3164 to control group. The weighted mean age was 66.7 ± 10.6 years. Females represented 34.4% of the study population. The follow-up periods ranged from 8 to 72 weeks. The mean left ventricular ejection fraction was 33.7 ± 8.8%, the mean haemoglobin level was 12.4 ± 1.8 g/dL, and the mean transferrin saturation was 18.9 ± 10.1%. The administered FCM doses varied between 500 mg and 2040 mg. The detailed baseline characteristics are reported in *Table 1*. A low risk of bias was observed in 8 included trials, and moderate concerns, mainly related to reporting of results and randomization process, were observed in 3 RCTs (Supplementary Fig. S2).

**Table 1:** Baseline characteristics of included studies

Study name	Inclusion criteria	Age (years) Mean ± SD	Females %	FCM (n)	Control (n)	Total (n)	LVEF (%) Mean ± SD	Hb (g/dl) Mean ± SD	TSAT (%) Mean ± SD	Dosage of iron therapy (mg)	Follow-up weeks	Main background medical therapy				
												ACEi/ARB/ARNI (%)	Beta blockers (%)	MRAs (%)	Loop Diuretic (%)	SGLT2 Inhibitor (%)
Anker 2025/FAIR-HF 2**^29^**	Patients with HF (LVEF ≤45%), iron deficiency (serum ferritin level <100 ng/mL; or if transferrin saturation was <20%, a serum ferritin level between 100 ng/mL and 299 ng/mL)	69.9 ± 11.7	33.3	558	547	1105	34 ± 8	12.5 ± 1.1	18.2 ± 9.2	0–12 months: 2040, 12–24 months: 925, 24–26 months: 750	72	95.8	90.3	69.2	82.6	23.3
Drozd 2024**^21^**	Men >18 y with HFrEF (LVEF ≤40%), NYHA I–III, iron deficiency, Hb 10–15; stable ≥1 month	65.6 ± 4.7	0	11	12	23	30 ± 2	13.6 ± 0.3	19.8 ± 2.1	500 or 1000	24	96	96	72	48	NA
Mentz 2023/HEART-FID**^22^**	Chronic HFrEF; LVEF ≤45%; iron deficiency; Hb 9–13.5; stable therapy ≥4 weeks	68.6 ± 11	33.8	1532	1533	3065	30.7 ± 7.2	12.5 ± 1.4	23.4 ± 10.8	0–12 months: 1809, 12–24 months: 481, 24–26 months: 420	52	89	92	56	96	7.7
Mollace 2022**^24^**	HFpEF (ESC 2021 criteria); diastolic dysfunction; ± iron deficiency	62.7 ± 9.3	46.87	32	32	64	53.9 ± 5.8	NA	NA	500	8	74	85	87	82	NA
Martens 2021/IRON-CRT**^25^**	HFrEF with CRT ≥6 mo; LVEF ≤45%; NYHA II–III; ferritin <100 or 100–300 + TSAT <20%; ≥98% BiV pacing	72.5 ± 10.5	34	37	38	75	33 ± 8	13.3 ± 1.2	18.8 ± 6	959	12	87	99	74	55	NA
Dhoot 2020/FCM-HF-IN**^23^**	Chronic HF with iron deficiency (ferritin <100 or 100–300 with TSAT <20%); adults ≥18; haemoglobin ≥9 g/dL	53 ± 10.5	42	35	35	70	24.9 ± 5	11 ± 4	NR	NR	12	NA	NA	NA	NA	NA
Ponikowski 2020/AFFIRM AHF**^26^**	Recent hospitalization for acute HF; LVEF <50%; iron deficiency (ferritin <100 or 100–299 with TSAT <20%)	71 ± 11	45	558	550	1108	33 ± 10	12.3 ± 1.6	15 ± 8	0–12 months: 1352	52	76	83	66	86	NA
Yeo 2018/PRACTICE-ASIA-HF**^27^**	Acute decompensated HF (any EF) after stabilization; age ≥21; able to perform 6MWT; iron deficiency = ferritin <300 if TSAT <20%	62.6 ± 10.4	22	24	25	49	39 ± 18	11.6 ± 1.9	16 ± 10	1000	12	100	74	56	87	NA
Van Veldhuisen 2017/EFFECT-HF**^28^**	Chronic HF with iron deficiency; peak VO₂ assessment; LVEF ≈32%; follow-up 24 weeks	63.5 ± 11.5	25	88	86	174	33 ± 9	12.9 ± 1.3	17	1204	24	94	98	67	93	NA
Ponikowski 2014/CONFIRM HF**^13^**	Chronic HFrEF; NYHA II–III; iron deficiency; impaired exercise tolerance	69.2 ± 9.4	47	150	151	301	37 ± 8	12.3 ± 1.4	20 ± 18	0–12 months: 1500	52	100	89	63	88	NA
Anker 2009/FAIR HF**^12^**	Chronic HFrEF; NYHA II–III; iron deficiency (ferritin <100 or 100–299 with TSAT <20%); Hb 9.5–13.5	67.7 ± 10.6	54	304	155	459	32 ± 6	11.9 ± 1.3	18 ± 13	0–6 months: 1850	24	91	71	63	83	NA

6MWT, 6-minute walk test; ACEi, angiotensin-converting enzyme inhibitor; AF, atrial fibrillation; ARB, angiotensin receptor blocker; ARNI, angiotensin receptor–neprilysin inhibitor; BiV, biventricular; BMI, body mass index; CAD, coronary artery disease; CKD, chronic kidney disease; CrCl, creatinine clearance; CRT, cardiac resynchronization therapy; CV, cardiovascular; EF/LVEF, ejection fraction/left ventricular ejection fraction; ESC, European Society of Cardiology; FCM, ferric carboxymaltose; GDMT, guideline-directed medical therapy; Hb, haemoglobin; HF, heart failure; HFpEF, heart failure with preserved ejection fraction; HFrEF, heart failure with reduced ejection fraction; IV, intravenous; LVEF, left ventricular ejection fraction; MI, myocardial infarction; MRA, mineralocorticoid receptor antagonist; NT-proBNP, N-terminal pro–B-type natriuretic peptide; NYHA, New York Heart Association; SD, standard deviation; TSAT, transferrin saturation; VO_2_/Peak VO_2_, oxygen consumption/peak oxygen consumption.

### Primary outcome

#### Recurrent HHF or cardiovascular mortality

**Figure 1:**
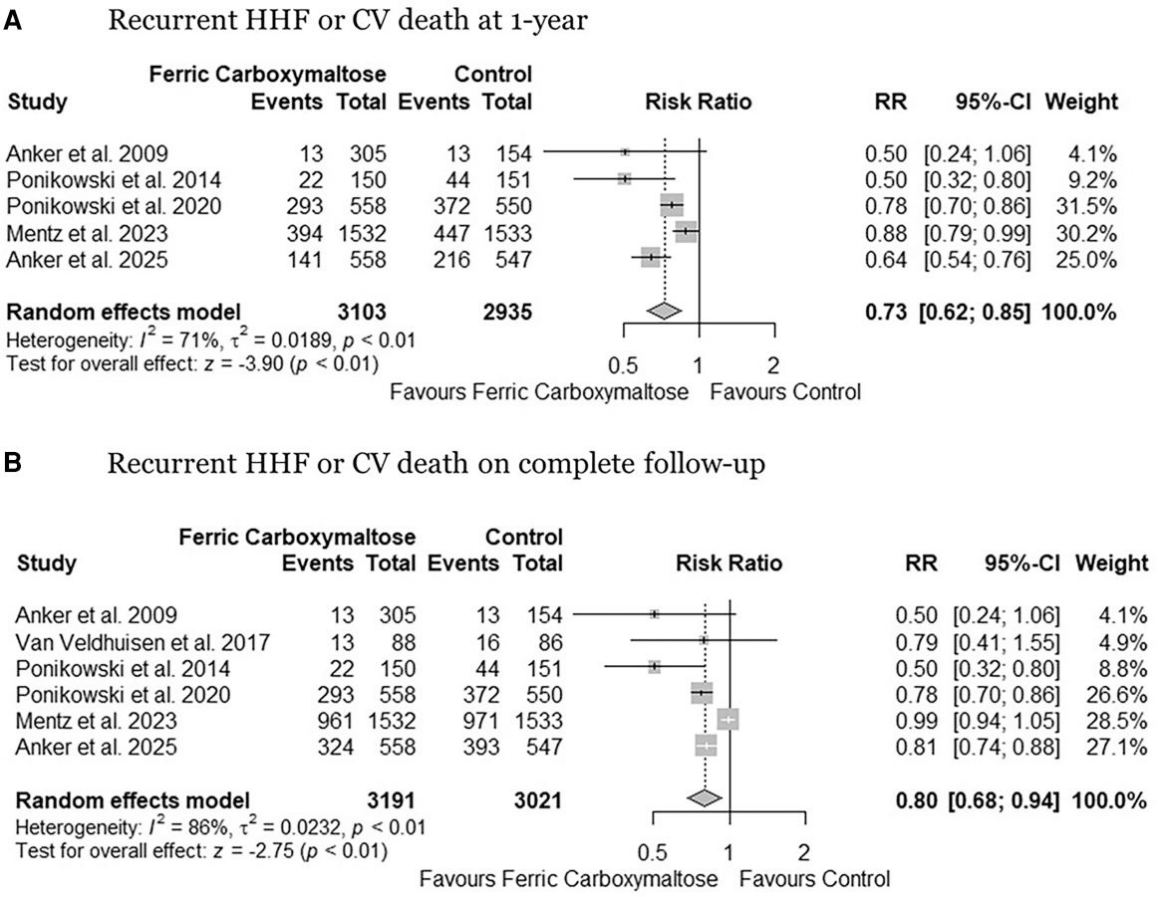
Forest plots for the (A) composite outcome of recurrent HHF or CV death 1 year and (B) complete follow-up. HHF, hospitalizations for heart failure; CV, cardiovascular

**Figure 2:**
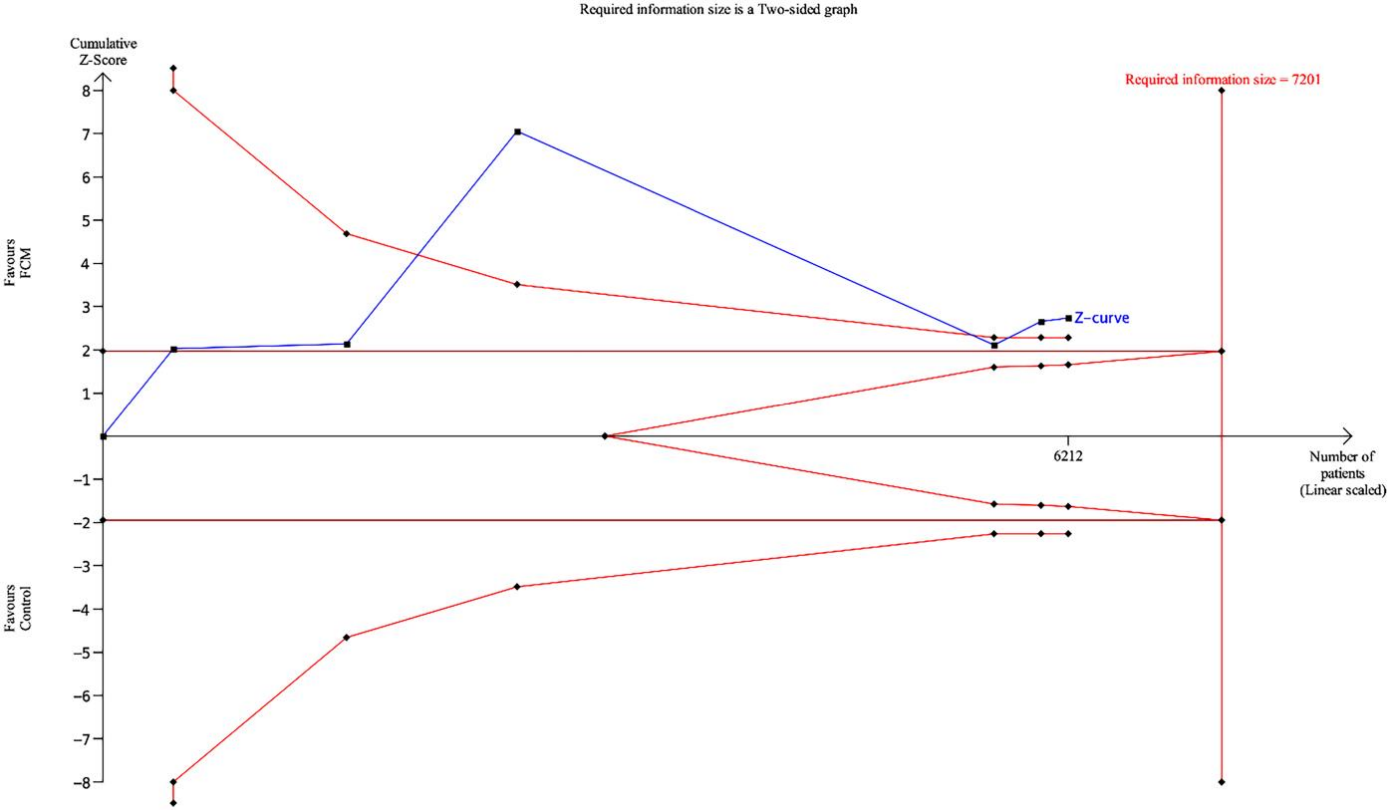
Trial sequential analysis for composite primary outcome. Trial Sequential Analysis (TSA) assessing the effect of intravenous ferric carboxymaltose (FCM) versus control on (recurrent HHF or cardiovascular death). The blue line represents the cumulative Z-curve of the included trials. The red inward-sloping lines denote the trial sequential monitoring boundaries, and the vertical red line indicates the required information size (RIS = 7201 patients). The cumulative Z-curve crossed the monitoring boundary before reaching the required information size, suggesting that firm evidence has been reached

The pooled analysis demonstrated that FCM administration was associated with a significantly reduced risk of recurrent HHF or CV mortality at 1-year follow-up (RR: 0.73, 95% CI: 0.62 to 0.85, *P* < .01, *Figure 1A*) (absolute risk reduction 100 (95% CI: 141 to 56) fewer per 1000 patients, high certainty, *Table 2*) and complete length of follow-up (RR: 0.80, 95% CI: 0.68 to 0.94, *P* < .01, *Figure 1B*) (absolute risk reduction 120 (95% CI: 192 to 36) fewer per 1000 patients, high certainty) compared to control group. Substantial heterogeneity (I^2^ = 71% at 1-year and I^2^ = 86% at complete follow-up) was observed. Heterogeneity reduced to 58% and 26% by excluding the HEART-FID trial^22^ from the pooled analysis (Supplementary Figs. S3 and S4). For the composite outcome of recurrent HHF or CVD death, the cumulative z-curve crossed both the conventional significance boundary and the trial sequential monitoring boundary, indicating that there is robust evidence supporting a potential 25% relative risk reduction with FCM compared to control (α-spending adjusted CI, 0.66 to 0.96, *Figure 2*).

**Table 2:** Summary of findings

Outcome	Patients (studies), N	Relative effect (95% CI)	Absolute effects (95% CI)			Certainty	What happens
			Control	FCM	Difference		
Recurrent HHF or CVD at complete follow-up	6212 (6 RCTs)	RR = 0.80 (0.68 to 0.94)	599 per 1000	479 per 1000 (407 to 563)	120 fewer per 1000 (from 192 fewer to 36 fewer)	⨁⨁⨁⨁ High	FCM reduces recurrent HHF or CVD at complete follow-up.
Recurrent HHF or CVD at 1 year	6038 (5 RCTs)	RR = 0.73 (0.62 to 0.85)	372 per 1000	272 per 1000 (231 to 316)	100 fewer per 1000 (from 141 fewer to 56 fewer)	⨁⨁⨁⨁ High	FCM reduces recurrent HHF or CVD at 1 year.
Recurrent HHF	6113 (6 RCTs)	RR = 0.75 (0.60 to 0.94)	456 per 1000	342 per 1000 (273 to 428)	114 fewer per 1000 (from 182 fewer to 27 fewer)	⨁⨁⨁⨁ High	FCM reduces recurrent HHF.
Recurrent HHF at 1 year	6038 (5 RCTs)	RR = 0.69 (0.57 to 0.84)	323 per 1000	223 per 1000 (184 to 271)	100 fewer per 1000 (from 139 fewer to 52 fewer)	⨁⨁⨁⨁ High	FCM reduces recurrent HHF at 1 year.
All-cause death at 1 year	6038 (5 RCTs)	RR = 0.86 (0.74 to 1.00)	111 per 1000	95 per 1000 (82 to 111)	16 fewer per 1000 (from 29 fewer to 0 fewer)	⨁⨁⨁◯ Moderate	FCM likely reduces all-cause death at 1 year slightly.
CVD at 1 year	6038 (5 RCTs)	RR = 0.86 (0.72 to 1.02)	84 per 1000	72 per 1000 (61 to 86)	12 fewer per 1000 (from 24 fewer to 2 more)	⨁⨁⨁◯ Moderate	FCM likely reduces CVD at 1 year.
All-cause death at complete follow-up	6406 (9 RCTs)	RR = 0.95 (0.86 to 1.05)	195 per 1000	185 per 1000 (168 to 205)	10 fewer per 1000 (from 27 fewer to 10 more)	⨁⨁⨁◯ Moderate	FCM likely reduces all-cause death at complete follow-up slightly.
CVD at complete follow-up	6406 (9 RCTs)	RR = 0.90 (0.79 to 1.02)	141 per 1000	127 per 1000 (111 to 144)	14 fewer per 1000 (from 30 fewer to 3 more)	⨁⨁⨁◯ Moderate	FCM likely reduces CVD at complete follow-up slightly.
6 MWT	4814 (7 RCTs)		0		29.19 (11.95 to 46.43)	⨁⨁⨁◯ Moderate	FCM likely increases 6 MWT.
Adverse events	5702 (6 RCTs)	RR = 1.05 (0.92 to 1.21)	456 per 1000	478 per 1000 (419 to 551)	23 more per 1000 (from 36 fewer to 96 more)	⨁⨁⨁◯ Moderate	FCM likely does not increase adverse events.

CI, confidence interval; CVD, cardiovascular death or cardiovascular event; FCM, ferric carboxymaltose; HHF, heart failure hospitalization; MD, mean difference; MWT, minute walk test; RCT, randomized controlled trial; RR, risk ratio.

### Secondary outcomes

#### Recurrent HHF

**Figure 3:**
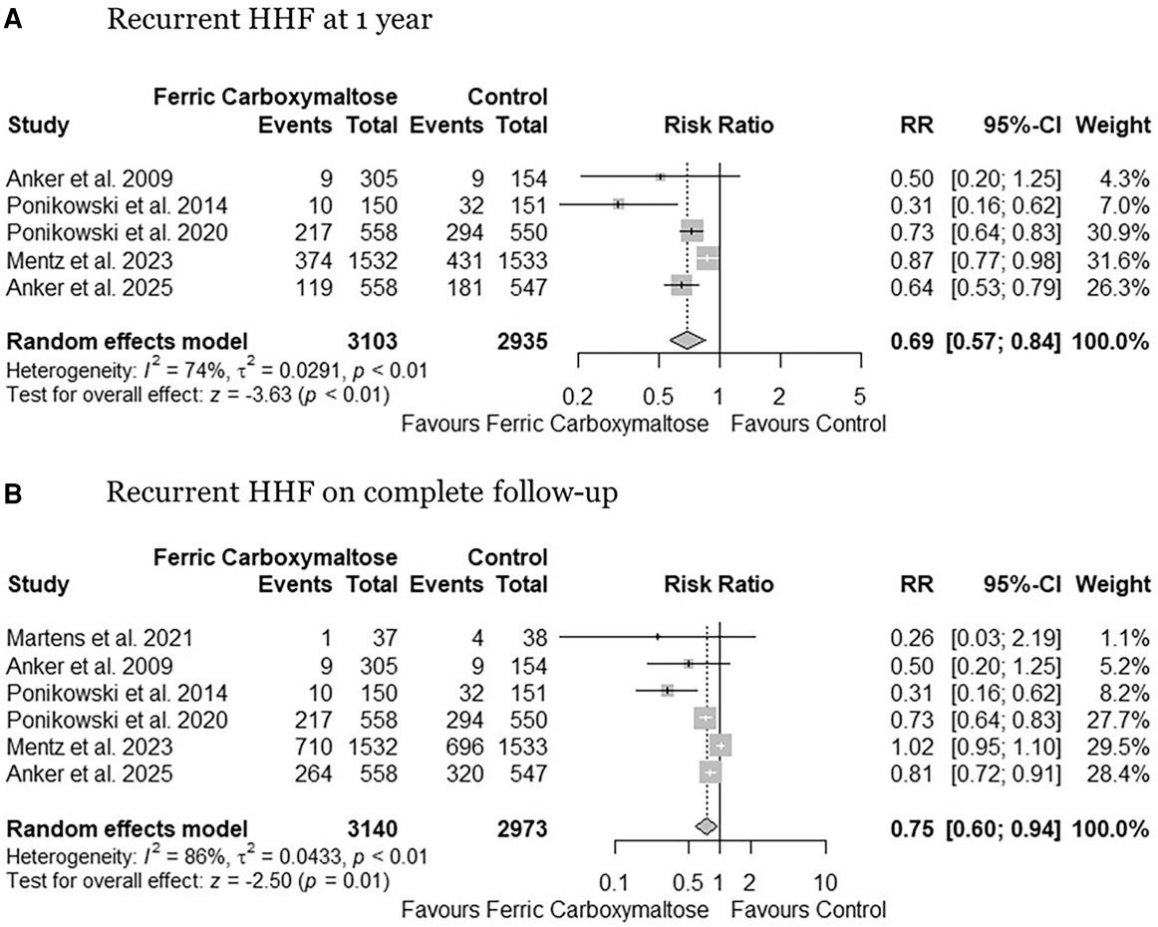
Forest plots for (A) recurrent HHF at 1 year, and (B) recurrent HHF on complete follow-up. HHF; hospitalizations for heart failure

Compared to the control group, FCM administration was associated with a significantly reduced risk of recurrent HHF at 1 year (RR: 0.69, 95% CI: 0.57 to 0.84, *P* < .01, *Figure 3A*) (absolute risk reduction 100 (95% CI: 139 to 52) fewer per 1000 patients, high certainty) and complete length of follow-up (RR: 0.75, 95% CI: 0.60 to 0.94, *P* < .01, *Figure 3B*) (absolute risk reduction 114 (95% CI: 182 to 27) fewer per 1000 patients, high certainty). A substantial heterogeneity (I^2^ = 74% at 1-year and I^2^ = 86% at complete follow-up) was observed. Heterogeneity reduced to 56% and 60% by excluding the HEART-FID trial^22^ from pooled analysis (Supplementary Figs. S5 and S6). For recurrent HHF (α-spending adjusted CI, 0.53 to 1.06, Supplementary Fig. S7), the cumulative z-score only crossed the conventional significance boundary, reflecting the need for further data to explore the impact of FCM on recurrent HHF.

#### Mortality

**Figure 4:**
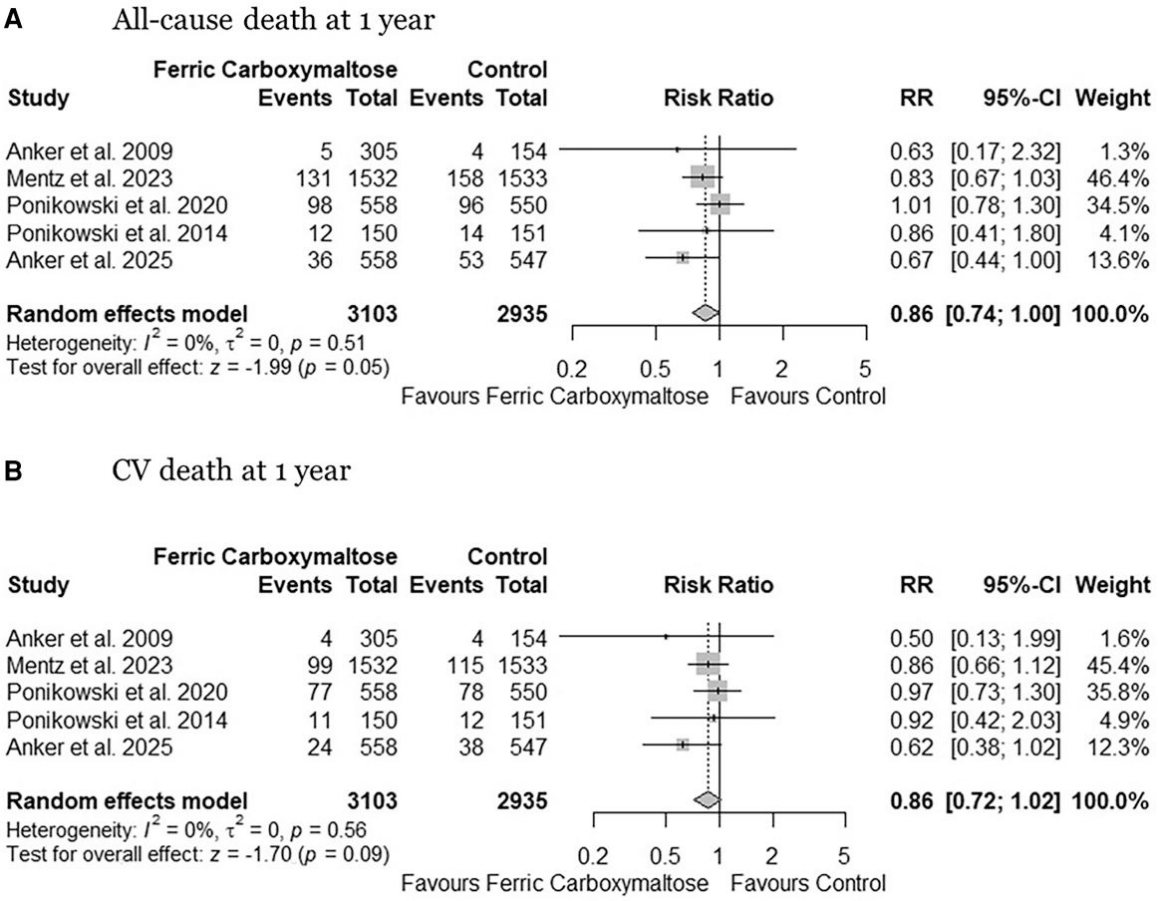
Forest plots for (A) All-cause death at 1-year and (B) CV death at 1-year. CV, cardiovascular

**Figure 5:**
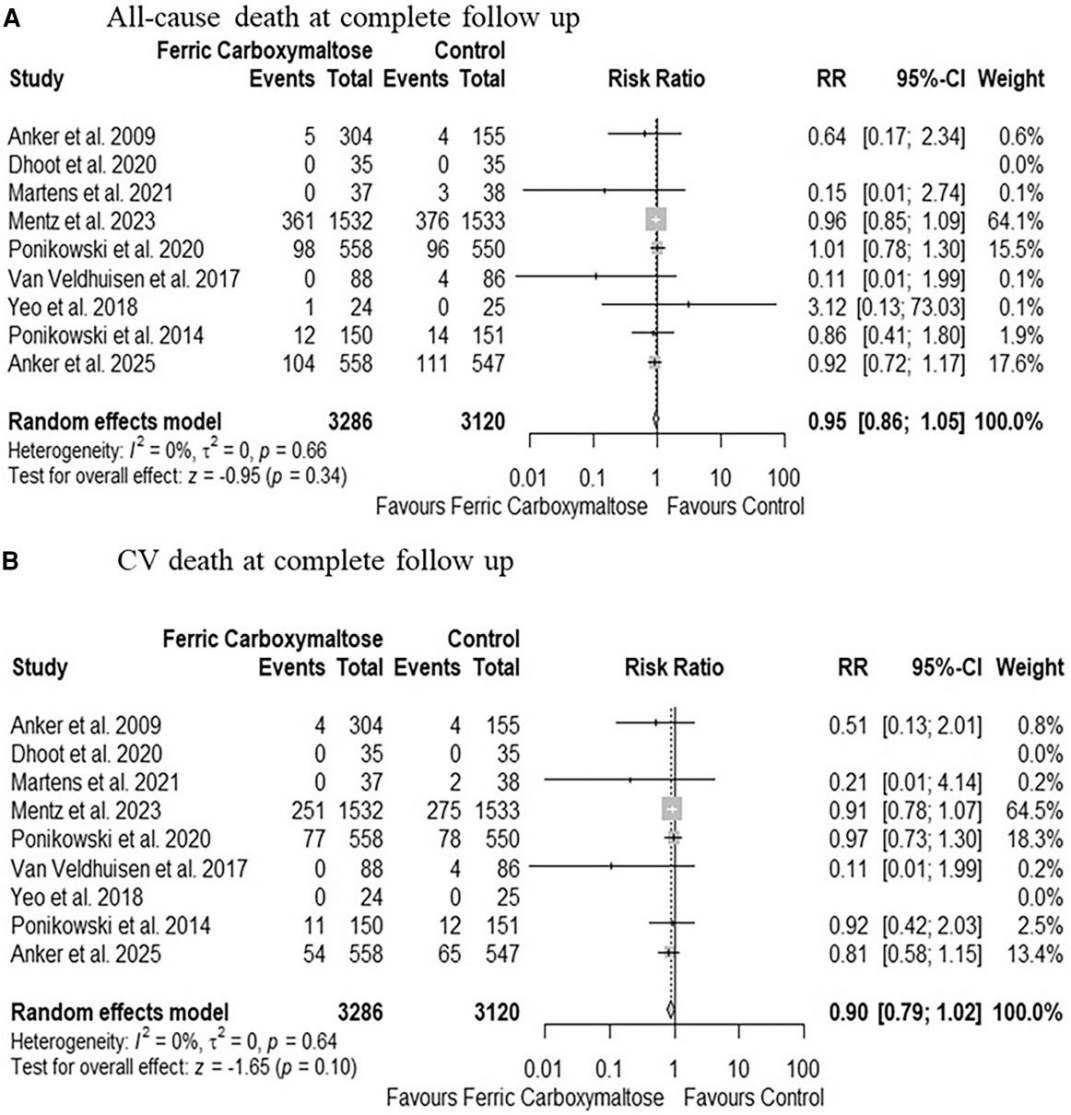
Forest plots for (A) All-cause death on complete follow-up and (B) CV death on complete follow-up. CV; cardiovascular

Compared to the control group, FCM administration showed a trend towards reduced risk of all-cause death (RR: 0.86, 95% CI: 0.74–1.00, *P* = .05, *Figure 4A*) (absolute risk difference 16 (95% CI 29 to 0) fewer per 1000 patients; moderate certainty) and CV death (RR: 0.86, 95% CI: 0.72–1.02, *P* = .09, *Figure 4B*) (absolute risk difference 12 (95% CI 24 fewer to 2 more) per 1000 patients; moderate certainty) at 1-year follow-up. However, over the complete follow-up period, the effect of FCM attenuated for both all-cause death (RR: 0.95, 95% CI: 0.86–1.05, *P* = .34, *Figure 5A*) (absolute risk difference 10 (95% CI 27 fewer to 10 more) per 1000 patients; moderate certainty) and CV death (RR: 0.90, 95% CI: 0.79–1.02, *P* = .10, *Figure 5B*) (absolute risk difference 14 (95% CI 30 fewer to 3 more) per 1000 patients; moderate certainty). No heterogeneity was observed (I² = 0%), and no outlier study was identified (Supplementary Figs. S8–S11). The cumulative z-curve for all-cause (α-spending adjusted CI, 0.85 to 1.07, Supplementary Fig. S12) and CV death (α-spending adjusted CI, 0.75 to 1.08, Supplementary Fig. S13) failed to cross the trial sequential monitoring boundaries, indicating a lack of firm evidence for a 25% reduction in all-cause and CV death with FCM compared with control.

#### 6-Minute walk test

Compared to the control group, FCM administration was associated with a significantly improved 6 MWT on follow-up (MD: 29.19 m, 95% CI: 11.95 to 46.43 m, *P* < .01, moderate certainty, Supplementary Fig. S14). A high heterogeneity was observed (I^2^ = 94%). No single outlier study was identified on leave-one-out sensitivity analysis (Supplementary Fig. S15).

#### Adverse events

Compared to control group, no statistically significant difference was observed regarding the risk of adverse events (including allergic reactions, headache, nausea, abdominal pain, or any other neurological, or gastrointestinal event) with FCM in patients with HF (RR: 1.02, 95% CI: 0.72 to 1.44, *P* = .93, moderate certainty, Supplementary Fig. S16). Heterogeneity was low (I^2^ = 43%) with no outlier study (Supplementary Fig. S17).

## Discussion

In this meta-analysis, we evaluated the effect of intravenous FCM in patients with HF and ID. Our study demonstrated with high certainty of evidence that IV treatment with FCM was associated with a significant reduction in the composite endpoint of recurrent HHF or CV death and recurrent HHF at 1-year and complete follow-up, compared with control. A trend towards lower risk of all-cause and CV death was observed in the FCM group at 1 year of follow-up; however, over the complete follow-up, the difference between the two groups attenuated (moderate certainty evidence). A significant improvement in the 6-MWT was also observed, reflecting better functional status in FCM-treated patients.

The observed reduction in the composite endpoint of recurrent HHF or CV death with FCM aligns with and reinforces previous research.^14,30^ This is likely attributed to FCM's ability to correct ID, improving exercise capacity by increasing peak oxygen consumption and enhancing aerobic performance, which is often impaired in HF patients with ID.^28^ Patients receiving FCM report significant improvements in symptoms and quality of life, as measured by the New York Heart Association (NYHA) functional class and Patient Global Assessment.^12,28^ These physiological improvements can translate into fewer hospital admissions and better overall CV outcomes.^31^ Importantly, our TSA findings confirmed that the cumulative evidence for the primary composite endpoint is robust suggesting that firm evidence exists for the benefit of FCM in reducing recurrent HHF or CV death. In contrast, the TSA for mortality outcomes did not cross the monitoring boundaries, indicating that additional adequately powered trials are still required to determine whether FCM can provide a survival benefit. This highlights that while the evidence for reducing hospitalizations is robust, uncertainty remains regarding mortality effects.

The trials often include patients with varying degrees of HF severity and different baseline characteristics, which can influence outcomes. For instance, the inclusion of the HEART-FID trial, the largest clinical trial to date evaluating intravenous FCM in patients with HFrEF and ID, had a significant impact on our pooled analysis.^6^ HEART-FID enrolled a relatively lower-risk population with high utilization of standard evidence-based medications, which may have attenuated the potential for additional benefit from iron repletion. Moreover, despite using the same ID definition, the baseline TSAT in HEART-FID was higher than other trials—potentially resulting in a distinct phenotype of ID. In contrast, the AFFIRM-AHF trial recruited patients during hospitalization for acute HF, focusing on those with recent acute episodes and therefore included a higher-risk cohort.^26^ These differing recruitment methodologies resulted in varying risk profiles among the studies, which may explain the differences in observed outcomes. The heterogeneity observed in the outcomes of recurrent HHF or CV death, and recurrent HHF specifically, is likely a result of this variability in patient characteristics, the severity of HF, and the timing of recruitment. Moreover, differences in ID definitions, dosing, and timing of FCM administration, and the conduct of the trial during the COVID-19 pandemic could have influenced clinical event rates, particularly hospitalization outcomes. This may explain why HEART-FID did not find a reduction in HHF over complete follow-up, unlike earlier trials.^22^ The recently published FAIR-HF2 trial further supports this, having enrolled ambulatory HFrEF patients but showing no significant reduction in CV death or first HHF, potentially due to high treatment discontinuation rates and a relatively short follow-up period influenced by the pandemic.^29^

In our meta-analysis, intravenous FCM was associated with a trend towards reduced all-cause and CV death at 1 year compared to control. However, this trend was not statistically significant, and the effect on mortality diminished with longer follow-up. The lack of observed mortality benefit may be attributed to factors such as the relatively short follow-up durations in these trials, the influence of the COVID-19 pandemic, and the inclusion of lower-risk populations, which could limit the ability to detect significant differences in mortality. Another plausible explanation is the early and complete correction of iron deficiency through high-dose FCM administered at the start of the trials. In later follow-up periods (>12 months), as seen in the HEART-FID and FAIR-HF2 trials, both dosing and treatment adherence declined. Participants received around 2000 mg of FCM in the first year, dropping to 300–900 mg annually thereafter. Notably, the initial IV iron dosing protocols varied across studies. Most trials concentrated FCM delivery within the first 4 to 6 weeks, with few patients receiving subsequent doses. This early intensive dosing likely contributed to the greater treatment effect observed in the first year. Sustained iron repletion, limited by COVID-19 disruptions, may have prolonged these benefits and warrants further investigation. However, it is important to note that the included trials were not specifically designed to test a dose–response relationship, and no direct evidence supports this interpretation. Thus, this should be viewed as a hypothesis-generating observation that warrants evaluation in future studies assessing long-term iron repletion strategies. Additionally, while our analysis could not assess subgroup differences such as sex due to lack of IPD, prior IPD-based analysis and post-hoc studies have suggested that men with HF may derive greater benefit from FCM compared to women.^15,32^ These findings highlight the need for future sex-stratified investigations to clarify potential treatment heterogeneity.

The present meta-analysis also evaluated the 6MWT, demonstrating a significant improvement in walk distance with FCM therapy compared to placebo, supporting the role of intravenous iron in enhancing exercise tolerance. Iron plays a crucial role in oxygen transport and mitochondrial energy production, both critical for skeletal and cardiac muscle function.^4^ In HF, ID can impair these processes, leading to reduced exercise capacity and increased fatigue.^13^ Intravenous iron therapy, such as FCM, can replenish iron stores, improve mitochondrial function, and enhance oxygen utilization, thereby improving exercise tolerance and quality of life in HF patients. Clinical trials have confirmed these findings, including the FAIR-HF trial demonstrated that FCM therapy improved symptoms, functional capacity, and quality of life in patients with chronic HF and ID, regardless of anaemia status.^12^ Although LVEF was not analysed as a pooled outcome due to limited and heterogeneously reported data (only IRON-CRT and Dhoot et al. provided results), both studies suggested potential improvements in cardiac function with FCM, supporting the hypothesis that iron repletion may enhance ventricular performance in addition to exercise capacity. Moreover, FCM is generally well-tolerated, with a favourable safety profile and no significant increase in major adverse events reported in HF populations.^33,34^ As emerging studies continue to elucidate the role of iron in HF, further trials are warranted to evaluate the comparative efficacy and safety of different iron formulations.

### Limitations

While this meta-analysis provides a comprehensive and current overview of the evidence, several limitations should be considered. First, as a study-level meta-analysis, the lack of patient-level data restricted our ability to account for individual effect modifiers such as ejection fraction, trial duration and patient severity, limiting the depth of analysis regarding patient-specific factors that may influence treatment outcomes. Second, there was variability in the inclusion criteria across the included trials, which may have introduced heterogeneity in the pooled results. Additionally, the substantial weight of the HEART-FID trial in this meta-analysis, due to its large sample size, may have disproportionately influenced the overall findings, potentially limiting the generalizability of the results to the broader HF population.

## Conclusion

IV FCM reduced the primary outcome of recurrent HHF or CV death in patients with HF and ID, with a consistent benefit observed in its components. FCM therapy led to a trend towards reduced 1-year mortality without reaching statistical significance. Improvements in functional status were also seen, as reflected by increased 6-MWT. These findings highlight the role of FCM in lowering the burden of hospitalizations and improving exercise tolerance in this population, though further research is needed to establish its effect on long-term survival.

## Supplementary Material

xvaf018_Supplementary_Data

## Declarations

### Data Availability

All data generated or analysed during this study are included in this article. Further inquiries can be directed to the corresponding author.

### Pre-registered Clinical Trial Number

None supplied.

## References

[1] Anand IS, Gupta P. Anemia and iron deficiency in heart failure: current concepts and emerging therapies. Circulation 2018;138:80–98. 10.1161/CIRCULATIONAHA.118.030099 29967232

[2] Cleland JGF, Zhang J, Pellicori P, Dicken B, Dierckx R, Shoaib A, et al Prevalence and outcomes of anemia and hematinic deficiencies in patients with chronic heart failure. JAMA Cardiol 2016;1:539–47. 10.1001/jamacardio.2016.1161 27439011

[3] Jankowska EA, von Haehling S, Anker SD, Macdougall IC, Ponikowski P. Iron deficiency and heart failure: diagnostic dilemmas and therapeutic perspectives. Eur Heart J 2013;34:816–29. 10.1093/eurheartj/ehs224 23100285 PMC3596759

[4] Alnuwaysir RIS, Hoes MF, van Veldhuisen DJ, van der Meer P, Grote Beverborg N. Iron deficiency in heart failure: mechanisms and pathophysiology. J Clin Med 2021;11:125. 10.3390/jcm11010125 35011874 PMC8745653

[5] Martens P, Nijst P, Verbrugge FH, Smeets K, Dupont M, Mullens W. Impact of iron deficiency on exercise capacity and outcome in heart failure with reduced, mid-range and preserved ejection fraction. Acta Cardiol 2018;73:115–23. 10.1080/00015385.2017.1351239 28730869

[6] Klip IT, Comin-Colet J, Voors AA, Ponikowski P, Enjuanes C, Banasiak W, et al Iron deficiency in chronic heart failure: an international pooled analysis. Am Heart J 2013;165:575–582.e3. 10.1016/j.ahj.2013.01.017 23537975

[7] Okonko DO, Mandal AKJ, Missouris CG, Poole-Wilson PA. Disordered iron homeostasis in chronic heart failure: prevalence, predictors, and relation to anemia, exercise capacity, and survival. J Am Coll Cardiol 2011;58:1241–51. 10.1016/j.jacc.2011.04.040 21903058

[8] Jankowska EA, Kasztura M, Sokolski M, Bronisz M, Nawrocka S, Oleśkowska-Florek W, et al Iron deficiency defined as depleted iron stores accompanied by unmet cellular iron requirements identifies patients at the highest risk of death after an episode of acute heart failure. Eur Heart J 2014;35:2468–76. 10.1093/eurheartj/ehu235 24927731

[9] Beattie JM, Khatib R, Phillips CJ, Williams SG. Iron deficiency in 78 805 people admitted with heart failure across England: a retrospective cohort study. Open Heart 2020;7:e001153. 10.1136/openhrt-2019-001153 32201585 PMC7066612

[10] McDonagh TA, Metra M, Adamo M, Gardner RS, Baumbach A, Böhm M, et al 2021 ESC guidelines for the diagnosis and treatment of acute and chronic heart failure. Eur Heart J 2021;42:3599–726. 10.1093/eurheartj/ehab368 34447992

[11] Heidenreich PA, Bozkurt B, Aguilar D, Allen LA, Byun JJ, Colvin MM, et al 2022 AHA/ACC/HFSA guideline for the management of heart failure: a report of the American College of Cardiology/American Heart Association Joint Committee on Clinical Practice Guidelines. Circulation 2022;145:e895–1032. 10.1161/CIR.0000000000001063 35363499

[12] Anker SD, Comin Colet J, Filippatos G, Willenheimer R, Dickstein K, Drexler H, et al Ferric carboxymaltose in patients with heart failure and iron deficiency. N Engl J Med 2009;361:2436–48. 10.1056/NEJMoa0908355 19920054

[13] Ponikowski P, van Veldhuisen DJ, Comin-Colet J, Ertl G, Komajda M, Mareev V, et al Beneficial effects of long-term intravenous iron therapy with ferric carboxymaltose in patients with symptomatic heart failure and iron deficiency. Eur Heart J 2015;36:657–68. 10.1093/eurheartj/ehu385 25176939 PMC4359359

[14] Ahmed M, Shafiq A, Javaid H, Singh P, Shahbaz H, Maniya MT, et al Intravenous iron therapy for heart failure and iron deficiency: an updated meta-analysis of randomized clinical trials. ESC Heart Fail 2025;12:43–53. 10.1002/ehf2.14905 38965691 PMC11769671

[15] Anker SD, Karakas M, Mentz RJ, Ponikowski P, Butler J, Khan MS, et al Systematic review and meta-analysis of intravenous iron therapy for patients with heart failure and iron deficiency. Nat Med 2025;31:2640–6. 10.1038/s41591-025-03671-1 40159279 PMC12353798

[16] Page MJ, McKenzie JE, Bossuyt PM, Boutron I, Hoffmann TC, Mulrow CD, et al The PRISMA 2020 statement: an updated guideline for reporting systematic reviews. BMJ 2021;372:n71. 10.1136/bmj.n71 33782057 PMC8005924

[17] Sterne JAC, Savović J, Page MJ, Elbers RG, Blencowe NS, Boutron I, et al Rob 2: a revised tool for assessing risk of bias in randomised trials. BMJ 2019;366:l4898. 10.1136/bmj.l4898 31462531

[18] DerSimonian R, Laird N. Meta-analysis in clinical trials. Control Clin Trials 1986;7:177–88. 10.1016/0197-2456(86)90046-2 3802833

[19] Higgins JPT, Thompson SG, Deeks JJ, Altman DG. Measuring inconsistency in meta-analyses. BMJ 2003;327:557–60. 10.1136/bmj.327.7414.557 12958120 PMC192859

[20] Wetterslev J, Jakobsen JC, Gluud C. Trial sequential analysis in systematic reviews with meta-analysis. BMC Med Res Methodol 2017;17:39. 10.1186/s12874-017-0315-7 28264661 PMC5397700

[21] Drozd MD, Tkaczyszyn M, Kasztura M, Węgrzynowska-Teodorczyk K, Flinta I, Banasiak W, et al Intravenous iron supplementation improves energy metabolism of exercising skeletal muscles without effect on either oxidative stress or inflammation in male patients with heart failure with reduced ejection fraction. Cardiol J 2024;31:300–8. 10.5603/cj.97253 37853824 PMC11076021

[22] Mentz RJ, Garg J, Rockhold FW, Butler J, De Pasquale CG, Ezekowitz JA, et al Ferric carboxymaltose in heart failure with iron deficiency. N Engl J Med 2023;389:975–86. 10.1056/NEJMoa2304968 37632463

[23] Dhoot S, Mittal S, Singh SP, Patel V, Kasliwal RR, Mehta V. Effect of ferric-carboxy maltose on oxygen kinetics and functional status in heart failure patients with iron deficiency. Future Sci OA 2020;6:FSO467. 10.2144/fsoa-2019-0156 32518682 PMC7273388

[24] Mollace A, Macrì R, Mollace R, Tavernese A, Gliozzi M, Musolino V, et al Effect of ferric carboxymaltose supplementation in patients with heart failure with preserved ejection fraction: role of attenuated oxidative stress and improved endothelial function. Nutrients 2022;14:5057. 10.3390/nu14235057 36501086 PMC9740330

[25] Martens P, Dupont M, Dauw J, Nijst P, Herbots L, Dendale P, et al The effect of intravenous ferric carboxymaltose on cardiac reverse remodelling following cardiac resynchronization therapy-the IRON-CRT trial. Eur Heart J 2021;42:4905–14. 10.1093/eurheartj/ehab411 34185066 PMC8691806

[26] Ponikowski P, Kirwan BA, Anker SD, McDonagh T, Dorobantu M, Drozdz J, et al Ferric carboxymaltose for iron deficiency at discharge after acute heart failure: a multicentre, double-blind, randomised, controlled trial. Lancet 2020;396:1895–904. 10.1016/S0140-6736(20)32339-4 33197395

[27] Yeo TJ, Yeo PSD, Hadi FA, Cushway T, Lee KY, Yin FF, et al Single-dose intravenous iron in Southeast Asian heart failure patients: a pilot randomized placebo-controlled study (PRACTICE-ASIA-HF). ESC Heart Fail 2018;5:344–53. 10.1002/ehf2.12250 29345426 PMC5880664

[28] Van Veldhuisen DJ, Ponikowski P, Van Der Meer P, Metra M, Böhm M, Doletsky A, et al Effect of ferric carboxymaltose on exercise capacity in patients with chronic heart failure and iron deficiency. Circulation 2017;136:1374–83. 10.1161/CIRCULATIONAHA.117.027497 28701470 PMC5642327

[29] Anker SD, Friede T, Butler J, Talha KM, Placzek M, Diek M, et al Intravenous ferric carboxymaltose in heart failure with iron deficiency: the FAIR-HF2 DZHK05 randomized clinical trial. JAMA 2025;333:1965. 10.1001/jama.2025.3833 40159390 PMC11955906

[30] Graham FJ, Pellicori P, Kalra PR, Ford I, Bruzzese D, Cleland JGF. Intravenous iron in patients with heart failure and iron deficiency: an updated meta-analysis. Eur J Heart Fail 2023;25:528–37. 10.1002/ejhf.2810 36823953 PMC10946839

[31] Rangwala BS, Zuhair V, Mustafa MS, Mussarat A, Khan AW, Danish F, et al Ferric carboxymaltose for iron deficiency in patients with heart failure: a systematic review and meta-analysis. Future Sci OA 2024;10:2367956. 10.1080/20565623.2024.2367956 38982752 PMC11238921

[32] Karakas M, Friede T, Butler J, Talha KM, Placzek M, Asendorf T, et al Intravenous ferric carboxymaltose in heart failure with iron deficiency (FAIR-HF2 DZHK05 trial): sex-specific outcomes. Eur J Heart Fail 2025. 10.1002/ejhf.3742 PMC12765045 40740027

[33] Abouzid M, Tanashat M, Khlidj Y, Abuelazm M, Ramadan A, Altobaishat O, et al Efficacy and safety of intravenous iron therapy in heart failure patients with iron deficiency: a systematic review and meta-analysis of randomized controlled trials. Eur Heart J 2024;45:ehae666.3278. 10.1093/eurheartj/ehae666.3278

[34] Padda I, Sebastian SA, Fabian D, Sethi Y, Johal G. The efficacy and safety of ferric carboxymaltose in heart failure with reduced ejection fraction and iron deficiency: an updated systematic review and meta-analysis of randomized controlled trials. Diseases 2024;12:339. 10.3390/diseases12120339 39727669 PMC11727542

