# Supplementary material for: Intravenous ferric carboxymaltose in patients with heart failure and iron deficiency: a systematic review and meta-analysis of randomized controlled trials with trial sequential analysis
Source: ESC Heart Fail. 2026 Jan 8;13(1):xvaf018. doi: 10.1093/eschf/xvaf018 (PMC13108314; doi:10.1093/eschf/xvaf018)
Supplement: xvaf018_Supplementary_Data [file xvaf018_supplementary_data.docx]

**Supplementary Appendix**

Table S1: Detailed search strings used for each database

| Database | Search string | Records retrieved |
| --- | --- | --- |
| PubMed/MEDLINE | ("ferric carboxymaltose"[All Fields] OR "FCM"[All Fields]) AND ("heart failure"[All Fields] OR "cardiac failure"[All Fields] OR "congestive heart failure"[All Fields] OR "HF"[All Fields] OR "HFrEF"[All Fields] OR "HFpEF"[All Fields]) | 289 |
| Embase | ('ferric carboxymaltose'/exp OR 'ferric carboxymaltose':ti,ab OR 'fcm':ti,ab)  AND ('heart failure'/exp OR 'heart failure':ti,ab OR 'cardiac failure':ti,ab OR 'congestive heart failure':ti,ab OR 'hfref':ti,ab OR 'hfpef':ti,ab) | 709 |
| Cochrane Library | ("ferric carboxymaltose" OR "FCM") AND ("heart failure" OR "cardiac failure" OR "congestive heart failure" OR "HF" OR "HFrEF" OR "HFpEF") | 152 |

Figure S1: PRISMA flowchart showing the screening and study selection process

Records removed *before screening*

Duplicate records removed (n = 357)

Records identified from

PubMed/MEDLINE (n = 289)

Embase (n = 709)

Cochrane Library (n = 152)

Total (n= 1,150)

**Identification**

Records screened

(n = 793)

Records excluded

(n = 711)

Reports sought for retrieval

(n = 82)

**Screening**

Reports assessed for eligibility

(n = 82)

Reports excluded:

Single-arm studies, reviews, editorials, and observational studies (n = 71)

Studies included in qualitative synthesis

(n = 11)

Studies included in quantitative synthesis

(n = 11)

**Included**

Figure S2: Risk of bias assessment for the included trials


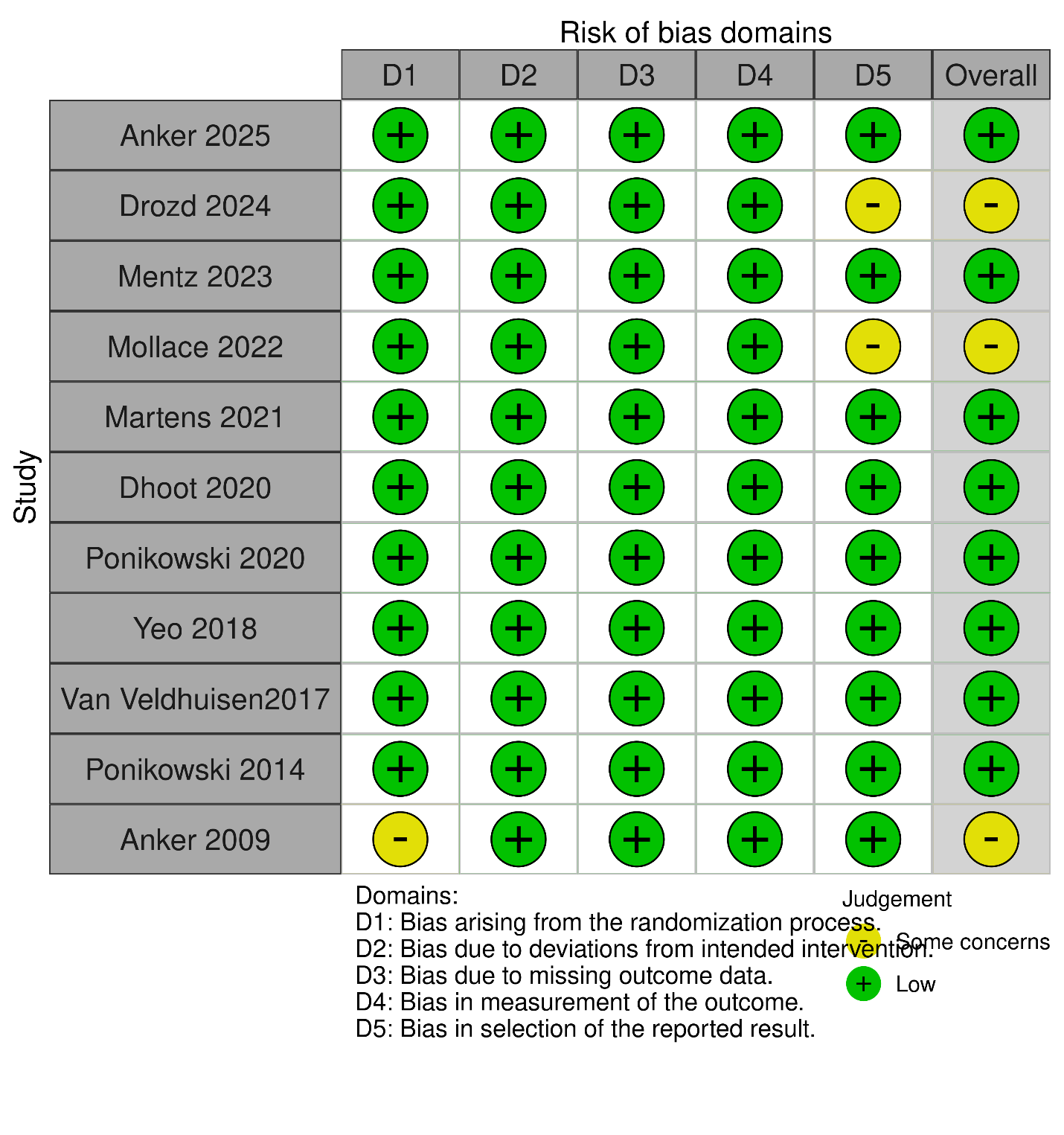


Figure S3: Leave-one-sensitivity analysis for composite primary outcome at 1-year follow-up


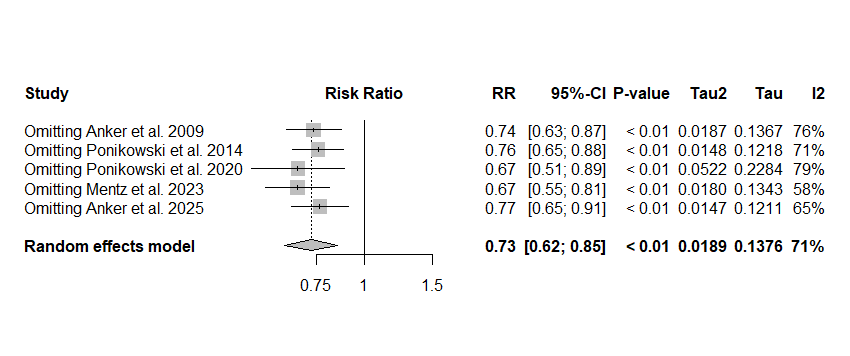


Figure S4: Leave-one-sensitivity analysis for composite primary outcome at complete follow-up


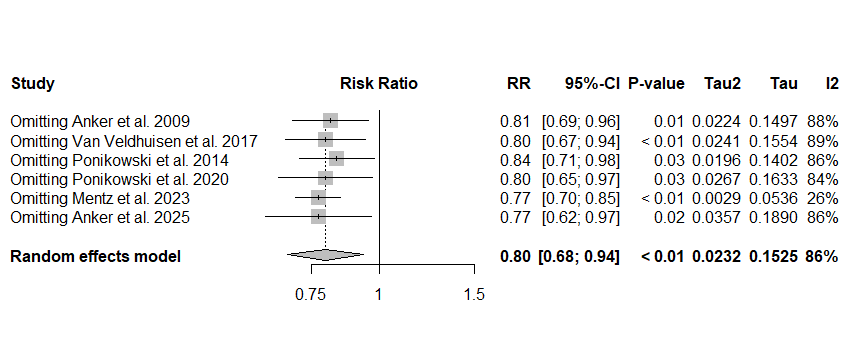


Figure S5: Leave-one-sensitivity analysis for recurrent HHF at 1-year follow-up


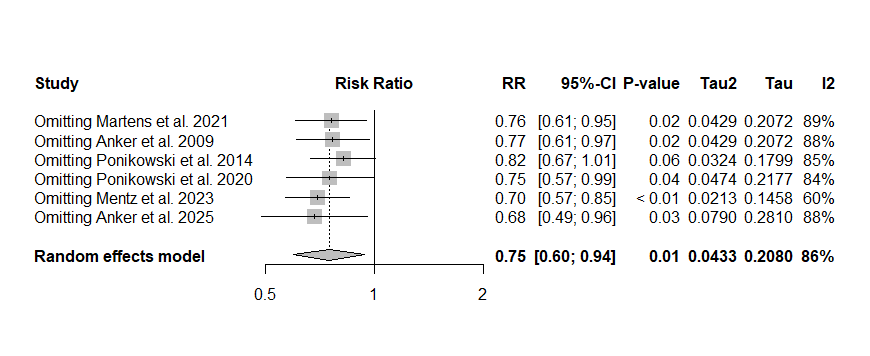


Figure S6: Leave-one-sensitivity analysis for recurrent HHF at complete follow-up


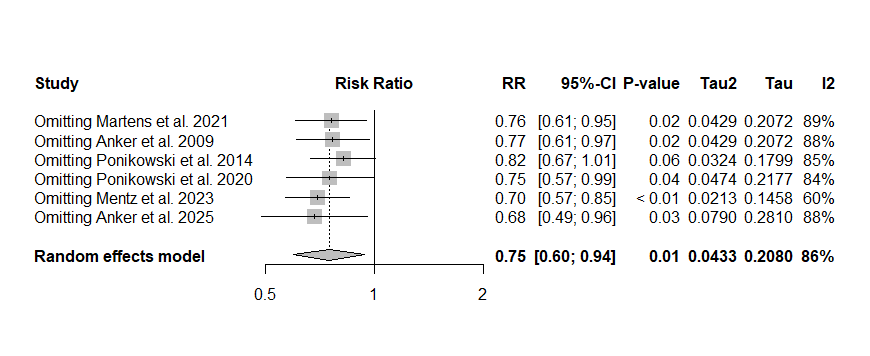


Figure S7: Trial sequential analysis for recurrent HHF


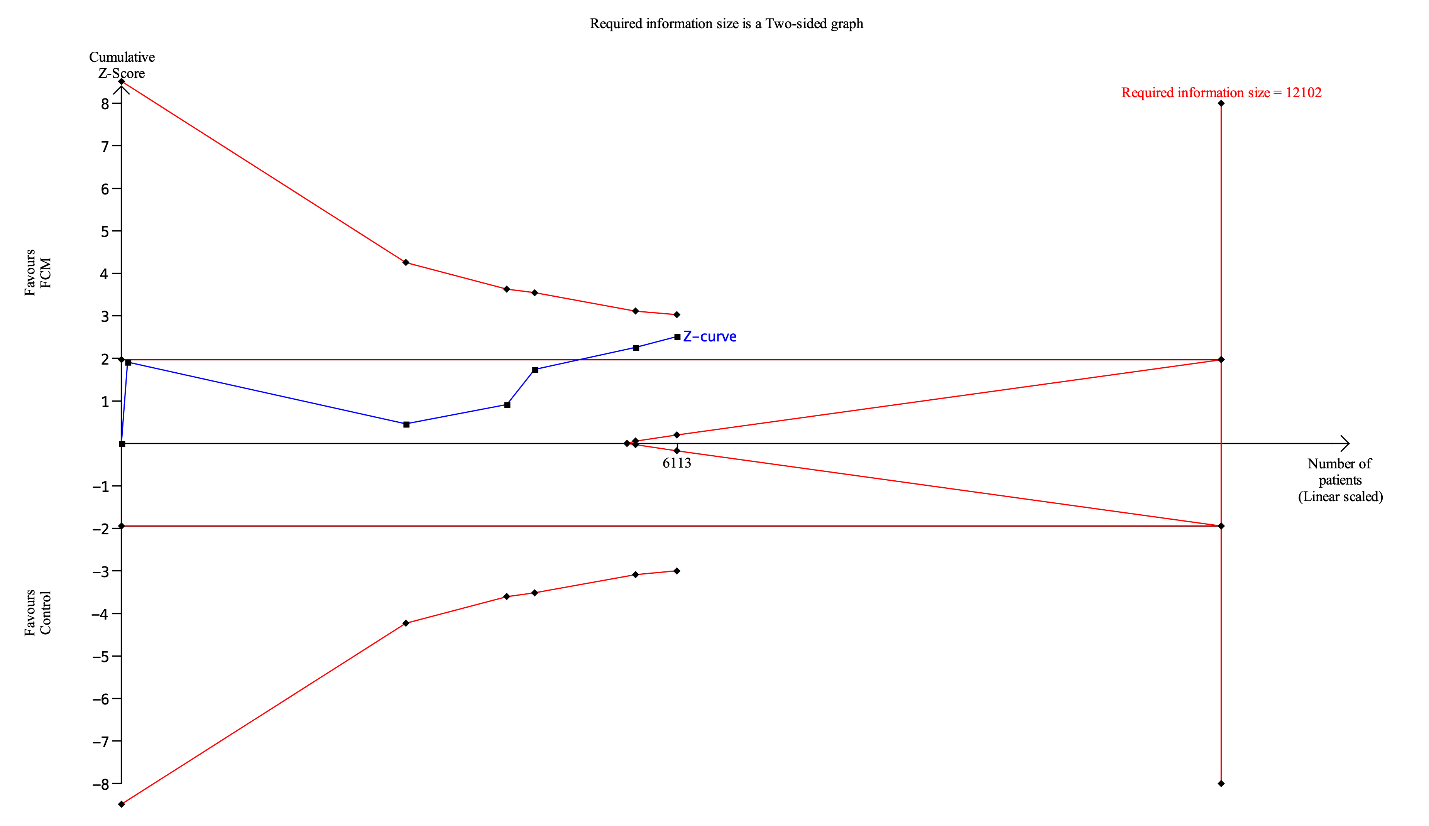


Figure S8: Leave-one-sensitivity analysis for all-cause death at 1-year


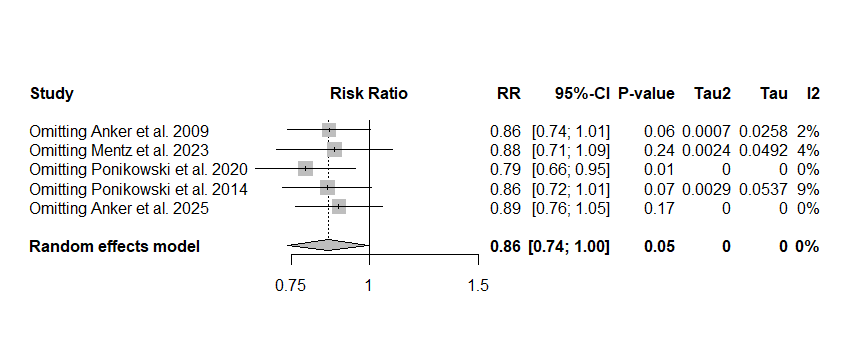


Figure S9: Leave-one-sensitivity analysis for CV death at 1-year


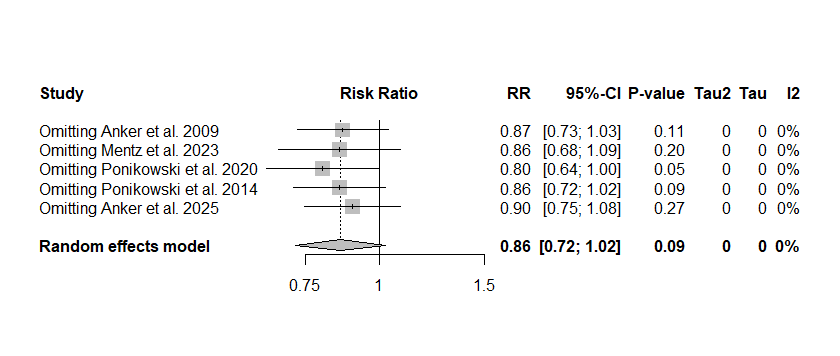


Figure S10: Leave-one-sensitivity analysis for all-cause death at complete follow-up


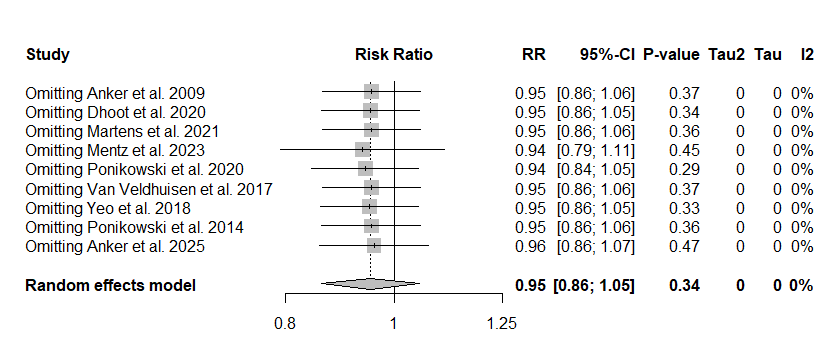


Figure S11: Leave-one-sensitivity analysis for CV death at complete follow-up


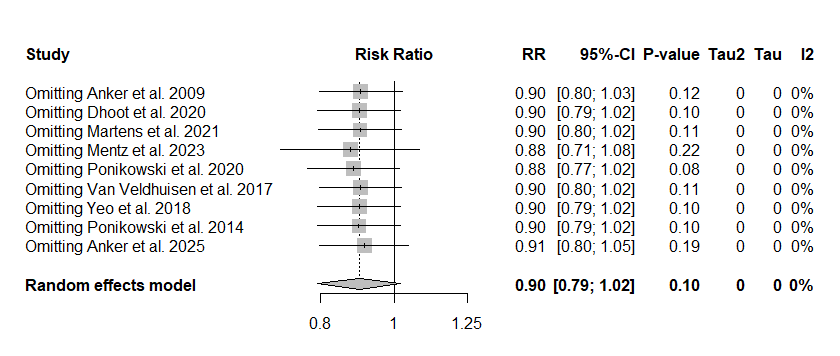


Figure S12: Trial sequential analysis for all-cause death


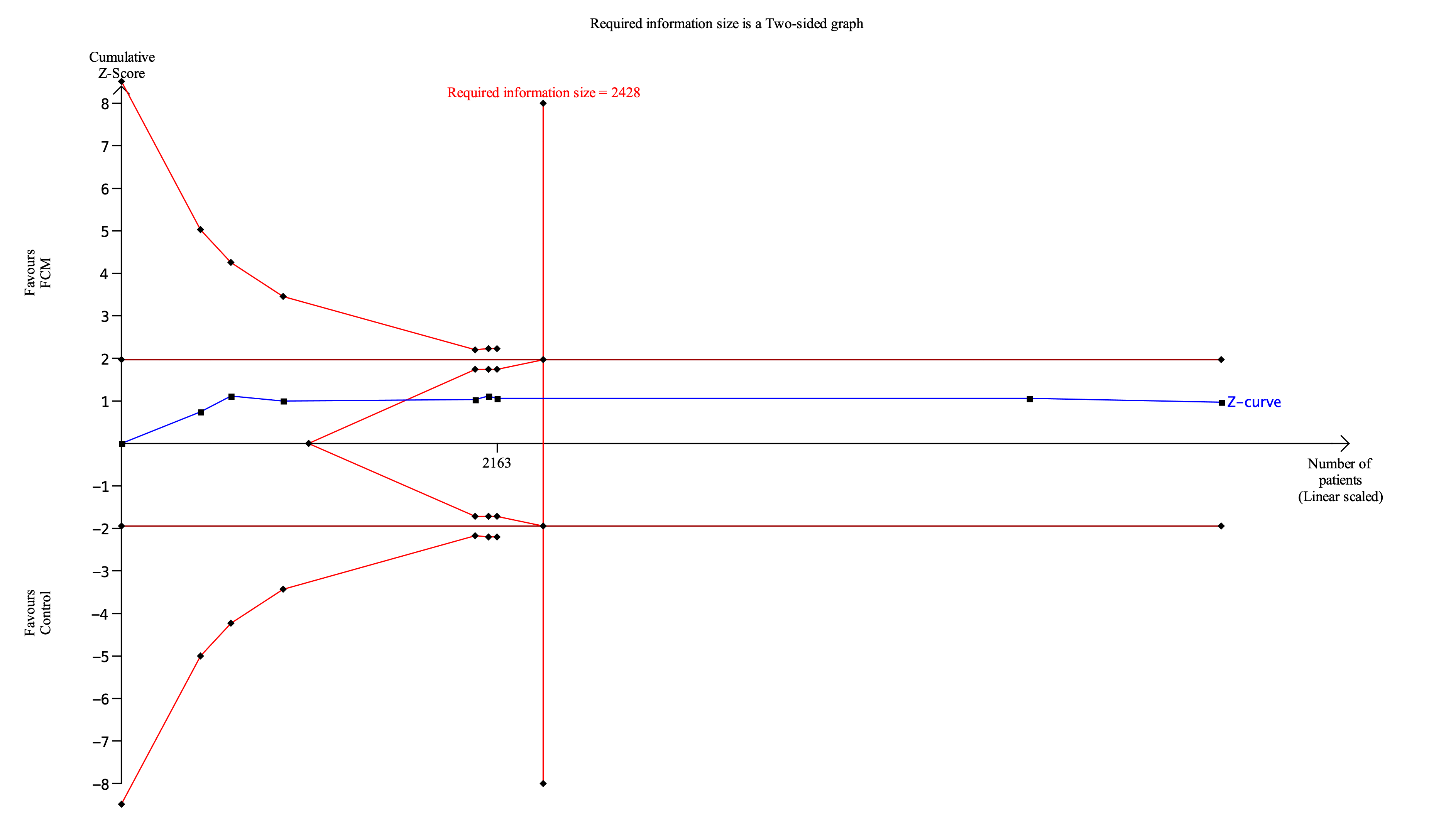


Figure S13: Trial sequential analysis for CV death


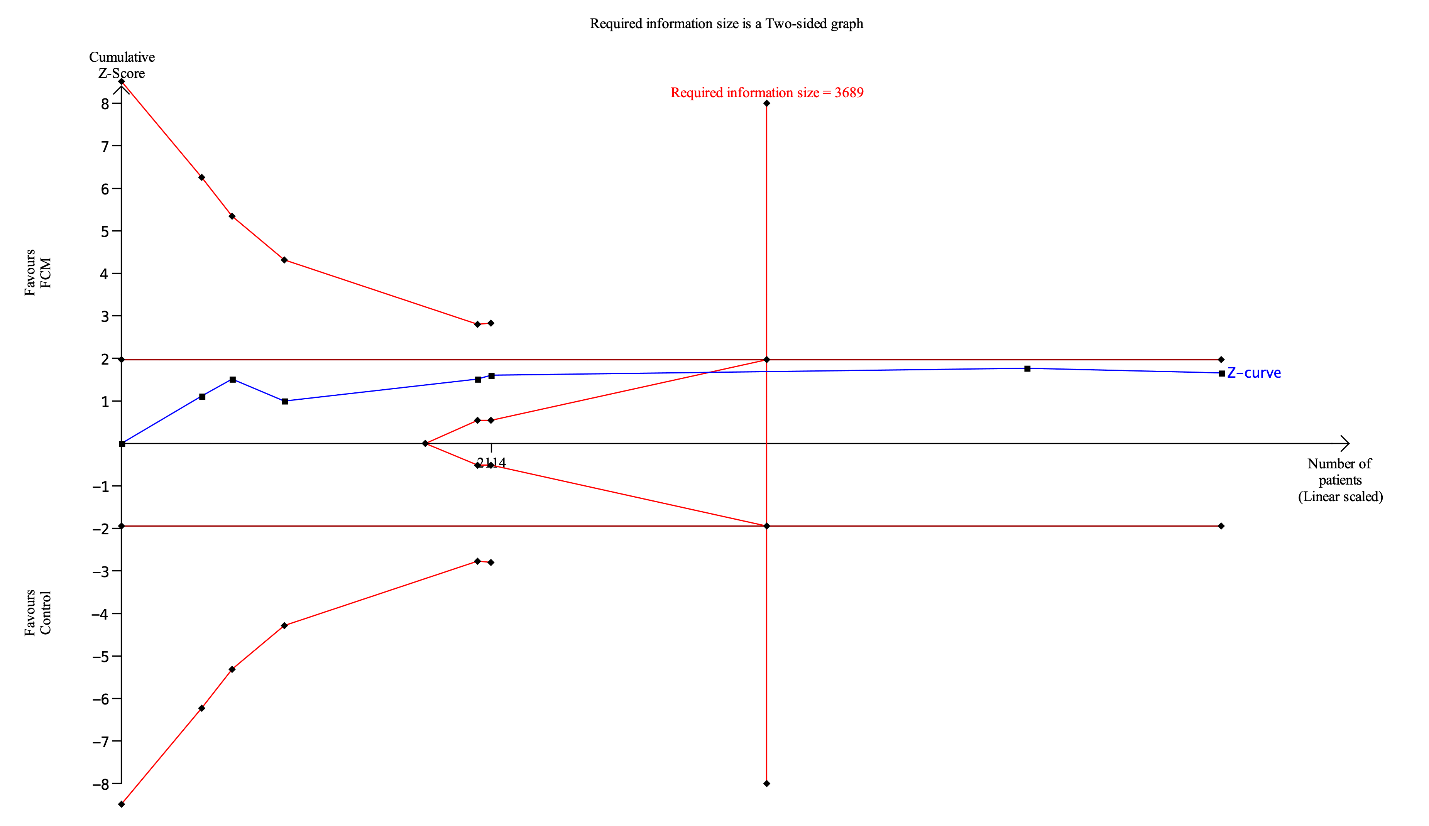


Figure S14: Forest plot for 6-minute walk time


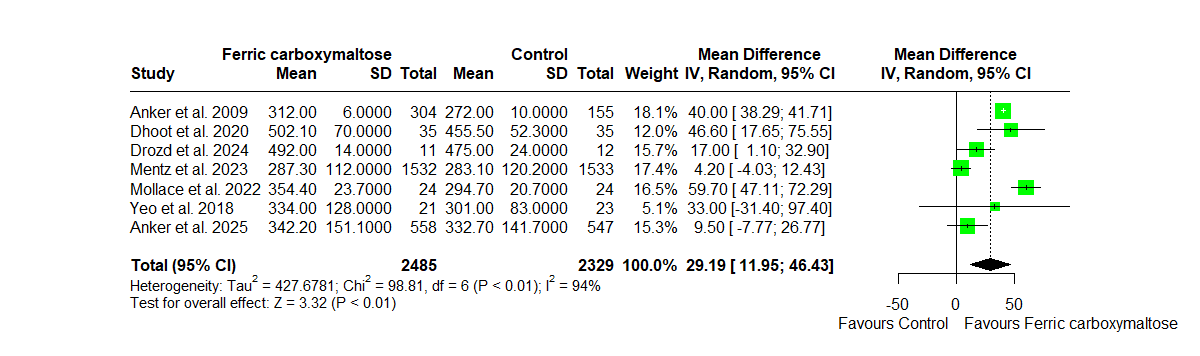


Figure S15: Leave-one-out sensitivity analysis for 6 minute walk time


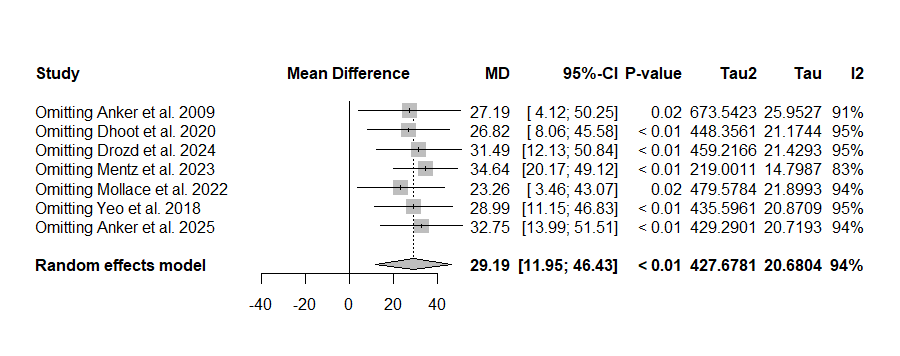


Figure S16: Forest plot for adverse events


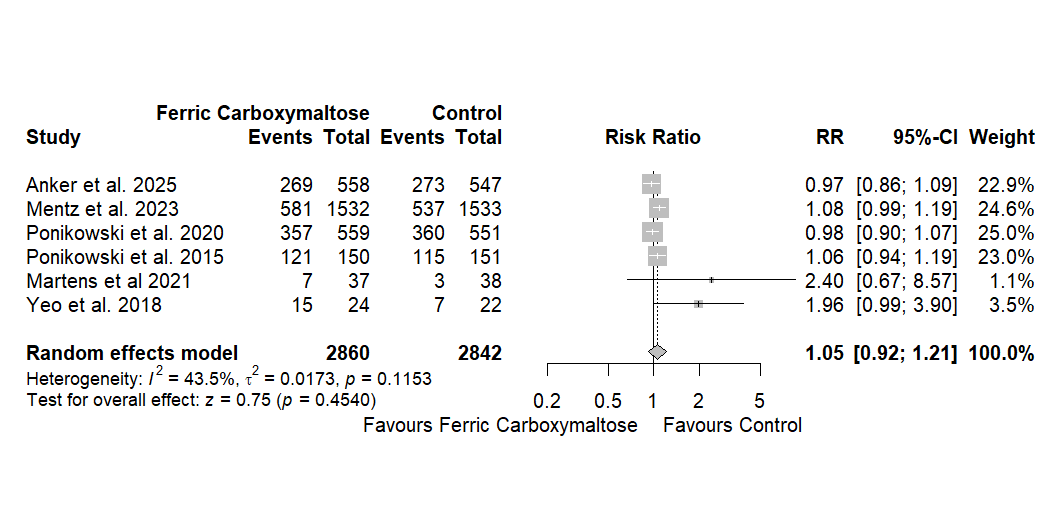


Figure S17: Leave-one-out sensitivity analysis for adverse events


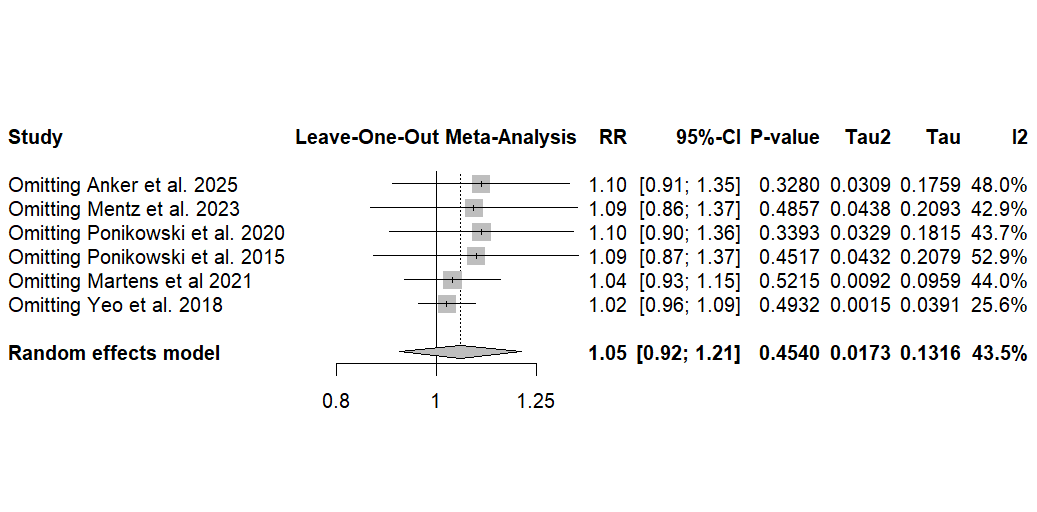


Table S2: PRISMA checklist

| **Section and Topic** | **Item #** | **Checklist item** | **Location where item is reported** |
| --- | --- | --- | --- |
| **TITLE** | | |  |
| Title | 1 | Identify the report as a systematic review. | Title page |
| **ABSTRACT** | | |  |
| Abstract | 2 | See the PRISMA 2020 for Abstracts checklist. | 4 |
| **INTRODUCTION** | | |  |
| Rationale | 3 | Describe the rationale for the review in the context of existing knowledge. | 5 |
| Objectives | 4 | Provide an explicit statement of the objective(s) or question(s) the review addresses. | 5-6 |
| **METHODS** | | |  |
| Eligibility criteria | 5 | Specify the inclusion and exclusion criteria for the review and how studies were grouped for the syntheses. | 7 |
| Information sources | 6 | Specify all databases, registers, websites, organisations, reference lists and other sources searched or consulted to identify studies. Specify the date when each source was last searched or consulted. | 6-7 |
| Search strategy | 7 | Present the full search strategies for all databases, registers and websites, including any filters and limits used. | Page 6, Supplemental appendix |
| Selection process | 8 | Specify the methods used to decide whether a study met the inclusion criteria of the review, including how many reviewers screened each record and each report retrieved, whether they worked independently, and if applicable, details of automation tools used in the process. | 7-8 |
| Data collection process | 9 | Specify the methods used to collect data from reports, including how many reviewers collected data from each report, whether they worked independently, any processes for obtaining or confirming data from study investigators, and if applicable, details of automation tools used in the process. | 7-8 |
| Data items | 10a | List and define all outcomes for which data were sought. Specify whether all results that were compatible with each outcome domain in each study were sought (e.g. for all measures, time points, analyses), and if not, the methods used to decide which results to collect. | 7-8 |
|  | 10b | List and define all other variables for which data were sought (e.g. participant and intervention characteristics, funding sources). Describe any assumptions made about any missing or unclear information. | 7-8 |
| Study risk of bias assessment | 11 | Specify the methods used to assess risk of bias in the included studies, including details of the tool(s) used, how many reviewers assessed each study and whether they worked independently, and if applicable, details of automation tools used in the process. | 8 |
| Effect measures | 12 | Specify for each outcome the effect measure(s) (e.g. risk ratio, mean difference) used in the synthesis or presentation of results. | 8-9 |
| Synthesis methods | 13a | Describe the processes used to decide which studies were eligible for each synthesis (e.g. tabulating the study intervention characteristics and comparing against the planned groups for each synthesis (item #5)). | 8-9 |
|  | 13b | Describe any methods required to prepare the data for presentation or synthesis, such as handling of missing summary statistics, or data conversions. |  |
|  | 13c | Describe any methods used to tabulate or visually display results of individual studies and syntheses. |  |
|  | 13d | Describe any methods used to synthesize results and provide a rationale for the choice(s). If meta-analysis was performed, describe the model(s), method(s) to identify the presence and extent of statistical heterogeneity, and software package(s) used. |  |
|  | 13e | Describe any methods used to explore possible causes of heterogeneity among study results (e.g. subgroup analysis, meta-regression). |  |
|  | 13f | Describe any sensitivity analyses conducted to assess robustness of the synthesized results. |  |
| Reporting bias assessment | 14 | Describe any methods used to assess risk of bias due to missing results in a synthesis (arising from reporting biases). | 8-9 |
| Certainty assessment | 15 | Describe any methods used to assess certainty (or confidence) in the body of evidence for an outcome. | 8 |
| **RESULTS** | | |  |
| Study selection | 16a | Describe the results of the search and selection process, from the number of records identified in the search to the number of studies included in the review, ideally using a flow diagram. | 9 |
|  | 16b | Cite studies that might appear to meet the inclusion criteria, but which were excluded, and explain why they were excluded. | 9 |
| Study characteristics | 17 | Cite each included study and present its characteristics. | 9 |
| Risk of bias in studies | 18 | Present assessments of risk of bias for each included study. | 9 |
| Results of individual studies | 19 | For all outcomes, present, for each study: (a) summary statistics for each group (where appropriate) and (b) an effect estimate and its precision (e.g. confidence/credible interval), ideally using structured tables or plots. | 9-11 |
| Results of syntheses | 20a | For each synthesis, briefly summarise the characteristics and risk of bias among contributing studies. | 10-11 |
|  | 20b | Present results of all statistical syntheses conducted. If meta-analysis was done, present for each the summary estimate and its precision (e.g. confidence/credible interval) and measures of statistical heterogeneity. If comparing groups, describe the direction of the effect. |  |
|  | 20c | Present results of all investigations of possible causes of heterogeneity among study results. |  |
|  | 20d | Present results of all sensitivity analyses conducted to assess the robustness of the synthesized results. |  |
| Reporting biases | 21 | Present assessments of risk of bias due to missing results (arising from reporting biases) for each synthesis assessed. | 9-10/Supplemntal Figure 2 |
| Certainty of evidence | 22 | Present assessments of certainty (or confidence) in the body of evidence for each outcome assessed. | Table 2 |
| **DISCUSSION** | | |  |
| Discussion | 23a | Provide a general interpretation of the results in the context of other evidence. | 11-12 |
|  | 23b | Discuss any limitations of the evidence included in the review. | 13-14 |
|  | 23c | Discuss any limitations of the review processes used. | 13-14 |
|  | 23d | Discuss implications of the results for practice, policy, and future research. | 13 |
| **OTHER INFORMATION** | | |  |
| Registration and protocol | 24a | Provide registration information for the review, including register name and registration number, or state that the review was not registered. | 6 |
|  | 24b | Indicate where the review protocol can be accessed, or state that a protocol was not prepared. | 6 |
|  | 24c | Describe and explain any amendments to information provided at registration or in the protocol. | 6 |
| Support | 25 | Describe sources of financial or non-financial support for the review, and the role of the funders or sponsors in the review. | None |
| Competing interests | 26 | Declare any competing interests of review authors. | 2-3 |
| Availability of data, code and other materials | 27 | Report which of the following are publicly available and where they can be found: template data collection forms; data extracted from included studies; data used for all analyses; analytic code; any other materials used in the review. | Public |
